# Mental Shopping Calculations: A Transcranial Magnetic Stimulation Study

**DOI:** 10.3389/fpsyg.2020.01930

**Published:** 2020-08-04

**Authors:** Michal Klichowski, Gregory Kroliczak

**Affiliations:** ^1^Faculty of Educational Studies, Adam Mickiewicz University, Poznan, Poland; ^2^Action and Cognition Laboratory, Faculty of Psychology and Cognitive Science, Adam Mickiewicz University, Poznan, Poland

**Keywords:** mathematical cognition, arithmetic operation, functional lateralization, posterior parietal cortex, transcranial magnetic stimulation study

## Abstract

One of the most critical skills behind consumer’s behavior is the ability to assess whether a price after a discount is a real bargain. Yet, the neural underpinnings and cognitive mechanisms associated with such a skill are largely unknown. While there is general agreement that the posterior parietal cortex (PPC) on the left is critical for mental calculations, and there is also recent repetitive transcranial magnetic stimulation (rTMS) evidence pointing to the supramarginal gyrus (SMG) of the right PPC as crucial for consumer-like arithmetic (e.g., multi-digit mental addition or subtraction), it is still unknown whether SMG is involved in calculations of sale prices. Here, we show that the neural mechanisms underlying discount arithmetic characteristic for shopping are different from complex addition or subtraction, with discount calculations engaging left SMG more. We obtained these outcomes by remodeling our laboratory to resemble a shop and asking participants to calculate prices after discounts (e.g., $8.80–25 or $4.80–75%), while stimulating left and right SMG with neuronavigated rTMS. Our results indicate that such complex shopping calculations as establishing the price after a discount involve SMG asymmetrically, whereas simpler calculations such as price addition do not. These findings have some consequences for neural models of mathematical cognition and shed some preliminary light on potential consumer’s behavior in natural settings.

## Introduction

Neuropsychological studies in patients and neuroimaging reports in healthy individuals show that the neural circuits for mental calculations form a complex network of interacting areas. They involve the parietal lobes, e.g., the bilateral intraparietal sulcus, the posterior subdivisions of the superior parietal lobules, and the left angular and supramarginal gyri of the inferior parietal lobule (Gobel et al., 2001; Naccache and Dehaene, 2001; Dehaene et al., 2003, 2004; Eger et al., 2003; Fias et al., 2003; Piazza et al., 2004; Pinel et al., 2004; Nieder, 2005; Sato et al., 2007; Andres et al., 2008; Brozzoli et al., 2008; Domahs et al., 2008, 2010; Kaufmann et al., 2008), as well as the temporal lobes, e.g., the posterior inferior temporal gyrus (Daitch et al., 2016; Hermes et al., 2017; Yeo et al., 2017; Pinheiro-Chagas et al., 2018, 2019) and even the left frontal lobe, with the Broca’s area and its vicinity (Schmithorst and Brown, 2004; Shuman and Kanwisher, 2004; Majerus et al., 2010). While it is still unknown what subdivision of this network is the most critical for mental arithmetic, there is some agreement that such an area should be located in the posterior parietal cortex (PPC) on the left (Dehaene et al., 2004; Nieder, 2005). However, recent studies also suggest that the right PPC may exert some role in such mental operations too (Arsalidou and Taylor, 2011; Knops and Willmes, 2014; Sokolowski et al., 2017; Mock et al., 2018). Indeed, there is evidence that the more difficult/complex the arithmetic task, the more profound the engagement of the right PPC (Fehr et al., 2008; Hamid et al., 2011; Vansteensel et al., 2014; Klichowski and Kroliczak, 2017; Mock et al., 2018; cf. Semenza and Benavides-Varela, 2017; Semenza et al., 2017). Whether or not these earlier findings are relevant for more down-to-earth tasks such as shopping arithmetic, especially discount calculations, still remains to be seen.

The key evidence to resolve this issue could come from the use of transcranial magnetic stimulation (TMS), in particular repetitive TMS (rTMS), because TMS makes it possible to demonstrate the causal role of a selected brain area in the operations in question. Meanwhile, rTMS has been used only in studies on simple mental calculations (i.e., based on single digits; Salillas et al., 2012; Maurer et al., 2015) that are far removed from such daily chores as calculating prices in consumers’ heads. To the best of our knowledge, only one rTMS study thus far has examined multi-digit mental addition (e.g., 23 + 26) and subtraction (e.g., 49–26), which are much closer to numbers that consumers are likely to operate on, showing an atypical lateralization of the rTMS effect (right > left) for the supramarginal gyrus (SMG), a part of PPC (Montefinese et al., 2017). This effect is probably related to the fact that in the case of complex mental calculations on rather unfamiliar or accidental numbers, the visual working memory is strongly burdened, and there is also a need for maintaining intermediate results, and therefore, increased attentional resources (Majerus et al., 2010; Fias et al., 2013; Sokolowski et al., 2017). In fact, such difficult, memory-based, processes are supported by the right SMG (Mock et al., 2018). However, it is not known whether or not the right SMG is involved in other types of complex arithmetic, e.g., calculations of sale prices that are characteristic of everyday human activities (Dastjerdi et al., 2013). An example is shopping, wherein for discounts one typically subtracts a percentage value from small multi-digit numbers, i.e., the ones with values after the decimal point (e.g., 8.80–50%).

Depending on the type of task, as well as on the conditions in which a given task is performed, different neural mechanisms might be invoked for disparate kinds of mental calculations (Curtis et al., 2016; Yusoff et al., 2016; Beller et al., 2018; Hickendorff et al., 2019). For example, complex addition or subtraction is based on mechanisms that are distinct from the ones for complex multiplication or division. Similarly, school math calculation may engage different neural resources from those used in a store. Indeed, different complex calculations can be associated with disparate neural processes (Li et al., 2018), and therefore, can differentially/asymmetrically engage different subdivisions of the relevant neural circuits (Yusoff et al., 2016; Hawes et al., 2019). Thereby, one cannot assume that in the case of sale price calculations the role of the right SMG will be the same as in the case of multi-digit mental addition or subtraction (Montefinese et al., 2017). In fact, operating on familiar numbers that are often seen on shop labels (e.g., −50% or −25%) – even though they are multi-digit, and therefore, complex – may not require right-hemisphere resources such as visual working memory to the extent than does operating on unfamiliar numbers (which put greater load on memory capacity, and consequently, require critical engagement of right SMG; Rosenberg-Lee et al., 2011; Bloechle et al., 2016; Nemati et al., 2017; Mock et al., 2018). Therefore, we hypothesized that the right, as compared to left SMG, might be less critical for calculations of sale prices characteristic for consumers’ daily behavior. We did not expect to see any differential engagement of SMG from the two hemispheres in our control task, that is, price addition.

The outcomes we obtained do not rule out that the right SMG is involved in complex mental calculation, but its greater engagement is not specific for all complex arithmetic. Indeed, in discount arithmetic its role is less pronounced and the left SMG dominates instead. While these findings may have some consequences for neural models of mathematical cognition too, they provide one of the first pieces of evidence unveiling the neural substrates critical for consumer’s behavior.

## Materials and Methods

Our study was assessed and approved by the local Ethics Committee for Research Involving Human Subjects (opinion #9/10/01/2018). As such, all procedures and manipulations were carried out in accordance with the principles of the Declaration of Helsinki 2013 and its recent amendments.

### Participants

Twenty healthy volunteers (14 women, age: 20–27, mean = 20.9, *SD* = 1.6) who took part in this study gave their written informed consents prior to participation. All participants had normal or corrected-to-normal visual acuity. As confirmed by the results of the revised version of the Edinburgh Handedness Inventory (Dragovic, 2004), nearly all volunteers (18) declared themselves as right-handed (*laterality quotient* = 93.6, *SD* = 16.3, *laterality score* = 60.0, *SD* = 12.4), whereas two of them were left-handed (*laterality quotient* = −58.3, *SD* = 58.9, *laterality score* = −37.5, *SD* = 38.9). All participants were reimbursed for their time and efforts, obtaining 50 PLN (approximately $13 USD) in the form of a gift card to redeem at a shop with books and CDs.

### Stimuli and Procedure

**Figure 1:**
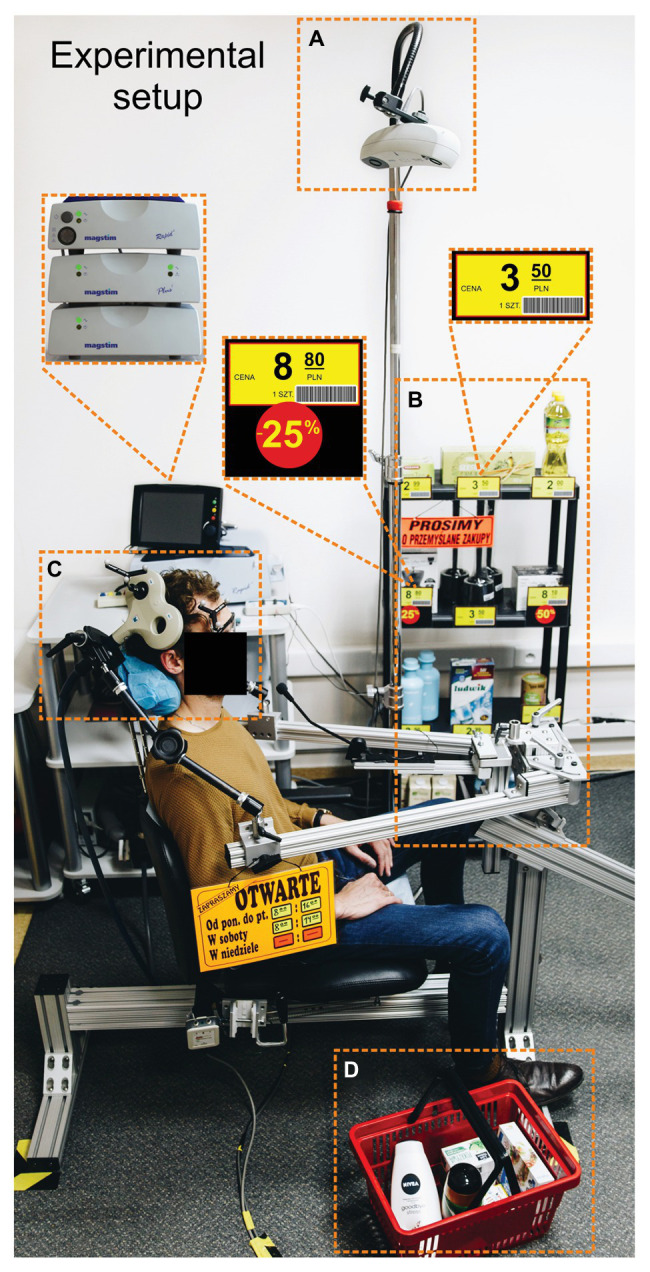
Experimental setup. **(A)** The tracking system for the head and coil. **(B)** A supermarket shelf. **(C)** One of the possible positions of the TMS coil (here over the right SMG). **(D)** A shopping basket. The translations of labels in Polish are the following: “cena” = “price,” “1 szt.” = “one piece,” “prosimy o przemyslane zakupy” = “please purchase carefully,” and “otwarte” = “open”.

To emulate the processes characteristic for shopping, we remodeled our laboratory so that it resembled a shop (see Figure 1). For example, at the entrance to the lab there was a shop sign with the opening hours. Next to the TMS chair, we put a shopping basket full of to-be-purchased items, and a supermarket shelf displaying different products and their prices.

**Figure 2:**
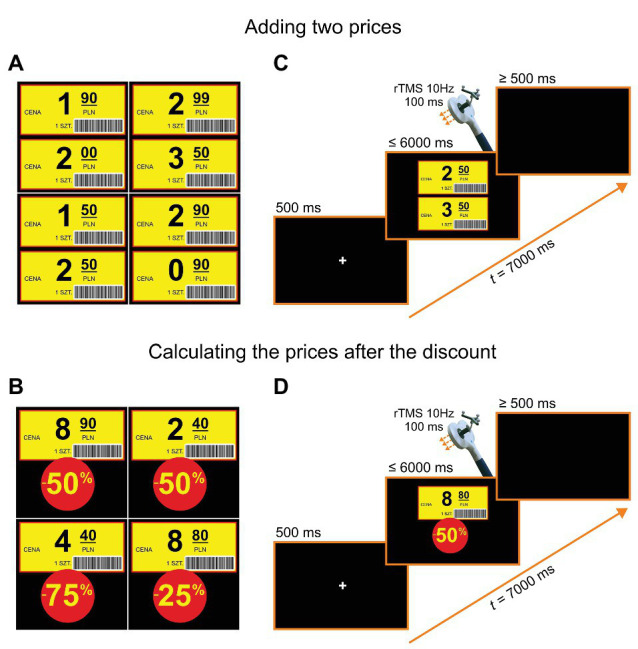
Example stimuli and trial structures. **(A)** Examples of stimuli for adding two prices. **(B)** Examples of stimuli for calculating the price after the discount. **(C)** Trial structure for adding two prices: In each trial, after a fixation point presented on a black screen for 500 ms, two shop labels were shown for a maximum of 6 s. After the onset of a participant’s vocal response, a black screen was introduced for a period of “6.5 s minus a response time” prior to the next trial (or in its absence, after a 6-s stimulus duration, for additional 500 ms). **(D)** Trial structure for calculating the prices after a discount: In each trial, after a fixation point presented on a black screen for 500 ms, one shop label and a discount value was shown for a maximum of 6 s (in the case of no response). As before, after the onset of a participant’s vocal response, a black screen was introduced for a period of 6.5 s minus a response time or minus stimulus duration. The translations of labels in Polish are the following: “cena” = “price”, “1 szt.” = “one piece”.

We used stimuli in the form of shop labels with numbers consisting of values with decimal points and values of discounts. Example stimuli are shown in Figures 2A,B. A participant’s task was to state the price after a discount (e.g., 8.80 PLN − 50% = 4.40 PLN, see Figure 2D), and in the control task, a sum of two prices (e.g., 2.50 PLN + 3.50 PLN = 6 PLN, see Figure 2C). The two tasks were presented in counterbalanced blocks of trials. The order of trials within blocks was pseudorandomized for different participants. Each block consisted of 40 trials, including 20 trials with rTMS. The whole study consisted of two sessions: one with stimulation over the right SMG and the other over the left SMG, with the order of stimulation sites counterbalanced across participants. Before the actual experiment, a noTMS training session (i.e., two blocks consisting of 10 trials) was administered. The stimuli used during the training session did not appear in the subsequent experiment.

A given stimulus was displayed on a 119.38 cm (47-inch) LG® TV screen (60 Hz, LG Electronics Inc., Seoul, Korea) and placed in front of the TMS chair at a distance of 2.28 m. Stimulus presentation was controlled by Cedrus® SuperLab for Windows version 4.5.4 (Cedrus® Corp., San Pedro, CA, USA) installed on a Dell® OptiPlex® 7010 computer (Dell Inc., Round Rock, TX, USA) and synchronized with the TMS stimulator using National Instruments PCI-DIO24 Digital I/O Card. Participants provided their answers vocally, and the experimenter constantly monitored their accuracies. Response times (RTs) – as measured by the onsets of vocal reactions – were detected by the SV-1 Smart Voice Key (Cedrus® Corp., San Pedro, CA, USA). A given stimulus was preceded by a fixation point in the form of a white cross over a black background and displayed for 500 ms. Following a response or after 6 s in the case of no response, a black screen was introduced between successive trials. Its duration was equal to 6.5 s minus RTs for the stimulus preceding it or 500 ms, if the participant did not provide an answer. Thus, the duration time for one trial was 7 s in total, but given that it was the experimenter who initiated the next trial only after confirming that the coil is still in the proper place, there was no risk of accumulation effects for subsequent series of stimulations (Rossi et al., 2009).

### rTMS Protocol

**Figure 3:**
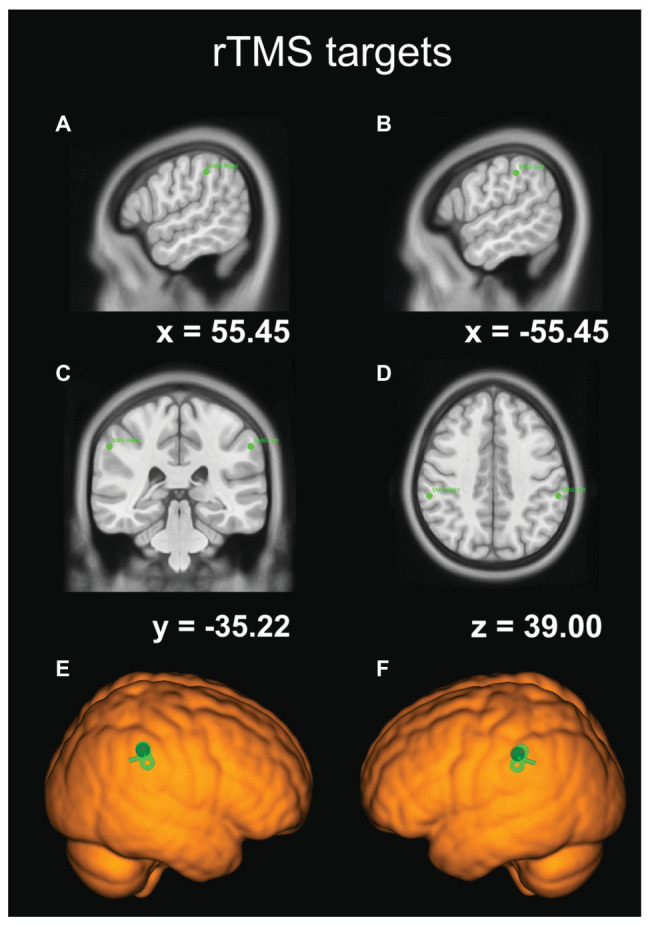
The coordinates of our rTMS targets in the Montreal Neurological Institute (MNI) template-brain space. **(A)** The sagittal view of the right SMG site. **(B)** The sagittal view of the left SMG site. **(C)** The frontal-plane view. **(D)** The transverse-plane view. **(E)** TMS target site rendered over the standard brain with TMS coil orientation for the right SMG. **(F)** TMS target site rendered over the standard brain with TMS coil orientation for the left SMG.

We adopted an rTMS protocol from an earlier study by Montefinese et al. (2017). Following the onset of a stimulus, namely at 100 ms, the rTMS train consisting of three (10 Hz) pulses – i.e., the first pulse at 100 ms, the second at 200 ms, and the last one at 300 ms – was delivered with a Magstim® Rapid 2 Plus 1 stimulator using a figure-of-eight coil (with the inner diameter of 70 mm). The angle of the coil relative to the mid-sagittal plane of the participant’s head was maintained at 45°, as it is the optimal coil orientation for stimulating PPC areas (Janssen et al., 2015). As in previous studies, a fixed stimulation intensity of 65% of the maximum stimulator output was used (for a review of the justifications for using fixed stimulation intensity, see Robertson et al., 2003; Vesia et al., 2010; Montefinese et al., 2017). Such stimulation protocol should result in a slowdown of RTs following rTMS. The TMS targets (the right and left SMG) were located with frameless stereotaxic neuronavigation (Brainsight® Frameless, Rogue Research Inc., Montreal, QC) and marked on the MNI ICBM 152 average brain template. Subsequently, participants’ brains were warped to this standard space with the Brainsight 2.3.9 registration algorithm for the MNI model head, utilizing five best landmarks, such as the frontmost, backmost, topmost, leftmost, and rightmost points on the skull in the scaling-step procedure. The coordinates of the SMG were selected based on previous studies (e.g., Potok et al., 2019; for details, see Figure 3). The positions of the coil and the head were constantly monitored with the Polaris® Vicra Optical Tracking System (Northern Digital Inc., Waterloo, ON, Canada) in real time (see Figure 1A). The coil was held tangentially and perpendicular to the surface of the scalp, its position was secured with a dual rod articulated arm (Manfrotto® 244N Variable Friction Magic Arm, Bassano del Grappa, Italy, see Figure 1C), and if necessary fine-tuned on-line by the experimenter. Moreover, to minimize head movements, participants’ heads were immobilized with the headrest and side support (see Figure 1).

### Data Analyses

In addition to comparisons between means, the main dependent variable in the critical analyses described below was the difference in RTs between rTMS over the left SMG and rTMS over the right SMG, referenced to noTMS baseline. In such comparisons, negative values indicate response facilitation and positive values indicate response delay following rTMS. The adopted level of significance was *α* = 0.05. For RTs accompanying correctly performed tasks, outliers greater than 6 s were discarded. All statistical analyses were carried out using IBM® SPSS Statistics® for Mac Version 26.0 (IBM Corp., Armonk, NY, USA). To compare RTs in disparate conditions and differences between rTMS effects over the left and right SMG, the following analyses were performed. Because the two tasks require quite different calculations, we ran two separate repeated-measures analyses of variance (rmANOVAs), one for adding prices and one for calculating discounts, with neural state (noTMS, rTMS-over-the-left-SMG, and rTMS-over-the-right-SMG) as the within-subjects factor. The necessary *post hoc* tests were performed with additional Bonferroni correction.

## Results

Neither the accuracy for adding prices nor calculation of discounts was affected by rTMS applied to the left or right SMG. Therefore, further analyses are limited to RTs for correctly performed calculations in each task.

**Figure 4:**
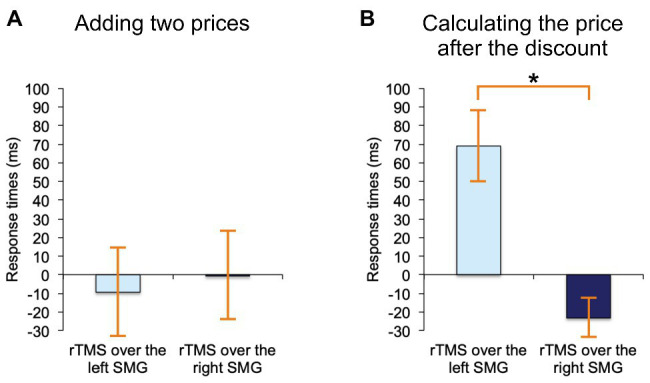
Results. **(A)** Adding two prices on shop labels: When we contrasted rTMS-over-the-left-SMG trials and rTMS-over-the-right-SMG trials, there were no significant differences in response times. **(B)** Calculating the prices after a typical shopping discount: When we contrasted rTMS-over-the-left-SMG trials with rTMS-over-the-right-SMG trials, there was a significant delay in response times following left SMG stimulations. Asterisk (*) indicates significant *p* (*p* < 0.05) and “0” the no-stimulation baseline. Error bars depict standards errors of the means.

The following rmANOVA for adding two prices revealed no significant effect whatsoever, regardless of the stimulation side (*F*_(2, 38)_ = 0.02, *p* = 0.98, see Figure 4A, where these results are expressed as difference scores, relative to noTMS baseline). Yet, for discount calculations rTMS affected RTs differently (*F*_(2, 38)_ = 3.6, *p* < 0.05), and the effect was such that only rTMS applied to the left SMG, as compared to right SMG, resulted in a significant slowdown of RTs (the difference between means = 92 ms, *SE* = 34 ms, *Bonferroni-corrected p* < 0.05). These results are, again, expressed as difference scores in Figure 4B. None of the results changed when the two left-handed participants were removed.

## Discussion

The outcomes of this study, based on the selected stimulation coordinates, as well as our shop-price label set, clearly show that it was the left SMG, as compared to its counterpart on the right, that was involved more (i.e., was asymmetrically engaged) in such shopping arithmetic as calculating discounts. The same type of stimulation, on the other hand, did not show any hemispheric asymmetries for adding prices. Discount calculation, a kind of task which is frequently performed by consumers before purchasing items in large superstores must, therefore, be dependent on a different involvement of key areas of the neural circuits underlying complex mental arithmetic (cf. Montefinese et al., 2017). One can only speculate that in less familiar settings and with less common prices/labels, a different asymmetric engagement of SMG would be revealed. Whether or not, under such circumstances, consumers are likely to calculate real prices after discounts, rather than trusting the sellers that there are bargains behind the “–XX%” labels, is an open question. Yet, consistent with some earlier evidence discussed above, if the size of the discount (the real price) was to be compared across different stores, the neural asymmetry for such processing could easily change its direction.

Importantly, our results indicate that the specific stimulation site of our choice, located in a rather anterior subdivision of the left SMG, plays a causal role in such highly complex arithmetic as discount price calculations. The greater engagement of left SMG, as compared to the right one, is consistent with a notion that this subdivision of PPC is also a part of the praxis representation network (PRN) for representing and sequencing highly complex and skilled actions in the typical brain (Przybylski and Kroliczak, 2017; see also Klichowski et al., 2020). After all, mathematical cognition is often linked to skilled manual/finger operations, and thus praxis (Rugani et al., 2017; Ras et al., 2019). Such embodiment of mental calculation may partly result from counting out loud and using fingers in learning arithmetic in childhood, when counting is a huge challenge (Brozzoli et al., 2008; Domahs et al., 2008). Because it takes longer for children to use fingers for more complex calculations than for less complex ones, therefore, the asymmetrically organized, more left-lateralized PRN can be a common framework for these types of mental arithmetic (Rapin, 2016).

Consistent with some of the above-mentioned arguments, it is quite unlikely that disparate kinds of mathematical, algebraic, or even shopping operations on price label contents or money, would be critically contingent on one specific mechanism, whether or not linked to SMG as a dominant node in a neural circuit. In fact, earlier research (e.g., Fias et al., 2003; Harvey et al., 2017; Kersey and Cantlon, 2017) shows the involvement of the intraparietal sulcus, the nearby subdivisions of the superior, as well as the inferior parietal lobule (including the angular gyrus) in different tasks involving numerosity processing and numbers. Thus, our null effect following right SMG TMS in consumers’ discount calculations does not preclude that more posterior or superior stimulation sites would reveal their causal contributions to such a task. Indeed, the outcomes of an earlier study by Montefinese et al. (2017) give pointers as to what kinds of calculations and stimulation sites should be used to disclose disparate neural mechanisms contributing to higher-level processing of numbers or supporting operations for them. Further questions to be also considered are differences in the timing when specific nodes of such a network contribute to disparate calculation tasks. It is, for example, quite possible that a selection of a different stimulation interval could interfere more with the discount-calculation task studied here. Whether or not this would also require different stimulation coordinates is yet another question to be addressed in future studies.

In sum, based on evidence from rTMS, our report demonstrates that left SMG, as compared to its right counterpart, plays a greater role in calculating prices after discounts. No effect of rTMS applied to left or right SMG was observed in our control task, that is, price addition. Yet, following study conclusion our participants claimed that TMS affected their ability to perform both tasks. This was clearly not the case. A change in protocol – including different timing of stimulation intervals, e.g., better correlated with proper chunking of responses leading to successful price calculations, would be required to show a casual contribution of SMG (especially SMG on the right) or other brain areas to both of the studied, but also related, tasks. While our positive findings add new knowledge to basic understanding of parietal lobe contributions to shopping mental calculations, further research is no doubt needed to elucidate the complexities of neural contributions to more sophisticated consumers’ behavior, both in and well beyond their self-reports.

## Data Availability Statement

The datasets generated for this study are available on request to the corresponding authors.

## Ethics Statement

The studies involving human participants were reviewed and approved by Ethics committee of Faculty of Psychology and Cognitive Science, Adam Mickiewicz University, Poznan, Poland. The patients/participants provided their written informed consent to participate in this study.

## Author Contributions

All authors listed have made a substantial, direct and intellectual contribution to the work, and approved it for publication.

### Conflict of Interest

The authors declare that the research was conducted in the absence of any commercial or financial relationships that could be construed as a potential conflict of interest.
